# Individual Comparative PK Evaluation of Single-dose Octocog Alfa, Rurioctocog Alfa Pegol, and Efanesoctocog Alfa in Adults with Severe Hemophilia A

**DOI:** 10.1055/a-2868-5490

**Published:** 2026-05-21

**Authors:** Janice M. Staber, Toshko Lissitchkov, Anthony K. C. Chan, Andrew S. Wilson, Jennifer Dumont, Annemieke J. Willemze

**Affiliations:** 1 Stead Family Department of Pediatrics, University of Iowa, Iowa City, Iowa, United States; 2 Department of Chemotherapy, Hemotherapy and Hereditary Blood Diseases, Clinical Hematology Clinic, Specialized Hospital for Active Treatment of Hematological Diseases, Sofia, Sofia, Bulgaria; 3 Department of Pediatrics, McMaster Centre of Transfusion Research, McMaster University, Hamilton, Canada; 4 74371 Sanofi, Cambridge, Massachusetts, United States; 5 Sanofi, Amsterdam, the Netherlands

People with severe hemophilia A experience frequent bleeds and subsequent joint damage and, therefore, rely on treatment with exogenous clotting factor VIII (FVIII) replacement or non-factor therapies. 1 FVIII replacement products are classified as either standard half-life (SHL) or extended half-life (EHL), although a universally accepted definition is currently lacking. 2 A variety of FVIII replacement products are currently available, but most bind von Willebrand factor (VWF) through noncovalent interactions, and therefore, their half-lives are limited by the chaperone effect of VWF. 3

Efanesoctocog alfa (Sanofi, Paris, France) is a first-in-class high-sustained FVIII replacement therapy composed of a single recombinant FVIII protein fused to dimeric fragment crystallizable (Fc) protein, 2 XTEN polypeptides, and the DʹD3 domain of VWF; 4 this fusion protein has been uniquely designed to decouple recombinant FVIII from endogenous VWF. As such, treatment with efanesoctocog alfa is not impacted by the VWF-imposed half-life ceiling and provides high-sustained FVIII activity. 5 6 7 Results from the phase 3 XTEND-1 study (NCT04161495) demonstrated that in adults and adolescents with severe hemophilia A, once-weekly efanesoctocog alfa prophylaxis (50 IU/kg) provided superior bleed protection versus pre-study FVIII prophylaxis, with normal to near-normal FVIII activity (>40%) for approximately 4 days, and 15% at day 7. 8 Results of a phase 1 sequential pharmacokinetic study demonstrated that an equivalent dose of efanesoctocog alfa had a half-life that was 3- or 4-fold longer, exposure (area under the FVIII activity–time curve [AUC]) that was 3- or 6-fold greater, and clearance that was 3.5- or 6-fold lower than that of corresponding values for EHL (rurioctocog alfa pegol) or SHL (octocog alfa) FVIII comparators, respectively. Furthermore, the observed coefficients of variation were lower for efanesoctocog alfa than comparator FVIII therapies. 9 Indeed, previous reports have described considerable interindividual pharmacokinetic variability among FVIII replacement products, 10 11 which may arise from interindividual differences in circulating VWF levels or VWF clearance rates during treatment. 9 12 13 14 Large interindividual pharmacokinetic variability inherently necessitates FVIII activity monitoring and dosing adjustments tailored to the individual, which, in turn, can increase healthcare resource utilization and associated costs. As efanesoctocog alfa is not impacted by endogenous VWF, its lower interindividual pharmacokinetic variability 9 may permit more accurate prediction of its FVIII activity profile across all patients, potentially reducing the need for intensive individual monitoring.

We conducted a complementary post hoc analysis of the aforementioned comparative phase 1 sequential pharmacokinetic study 9 to describe pharmacokinetic parameters for efanesoctocog alfa, and SHL (octocog alfa) and EHL (rurioctocog alfa pegol) FVIII therapies at the individual participant level. The design of this phase 1, single-center, single-arm, nonrandomized, open-label, fixed-sequence pharmacokinetic study (NCT05042440) has been previously reported. 9 All participants provided written informed consent before enrollment. The study was conducted in accordance with ethical principles derived from international guidelines, including the Declaration of Helsinki, the Council for International Organizations of Medical Sciences International Ethical Guidelines, applicable International Conference on Harmonization Good Clinical Practice guidelines, and applicable local laws and regulations. Briefly, the study cohort enrolled 13 adult males with severe hemophilia A (endogenous FVIII activity <1%) who had ≥150 exposure days of prior FVIII therapy. Key exclusion criteria were other known coagulation disorder(s), history of hypersensitivity or anaphylaxis to any FVIII product, history of a positive inhibitor test, and positive inhibitor result at screening. Eligible participants underwent screening for up to 28 days, including a washout period of either ≥4 or ≥5 days for participants whose most recent FVIII replacement was an SHL or EHL FVIII product, respectively. Participants then entered a 3-day octocog alfa treatment period, during which they each received a single dose of octocog alfa on the first day, followed by pharmacokinetic measurements and a 1-day washout period. This was followed by a 5-day rurioctocog alfa pegol treatment period during which participants received a single dose of rurioctocog alfa pegol on the first day, followed by pharmacokinetic measurements and a 2-day washout period. Lastly, participants entered a 14-day efanesoctocog alfa treatment period, during which they received a single dose of efanesoctocog alfa on the first day, followed by pharmacokinetic measurements and a 14-day observation period. All treatments were administered at a dose of 50 IU/kg. The primary objective of this complementary post hoc analysis was to assess the half-life of octocog alfa, rurioctocog alfa pegol, and efanesoctocog alfa for each participant after a single intravenous injection of each treatment. Secondary objectives included individual participant characterization of additional pharmacokinetic parameters for the three FVIII replacement products, including clearance, area under the curve extrapolated to infinity (AUC _0–inf_ ), and time above 10, 15, and 40% FVIII activity. Pharmacokinetic parameters were computed and analyzed by a noncompartmental method (Phoenix WinNonlin, version 8.1) using baseline-corrected FVIII activity. FVIII activity was measured using the one-stage activated partial thromboplastin time–based clotting assay with Actin FSL and validated using human plasma FVIII standard. FVIII activity and pharmacokinetic parameters were summarized by participant and treatment group using descriptive statistics.

Demographic and baseline characteristics and safety results from this phase 1 study have been previously reported. 9 Briefly, the population ( *n* = 13) had a mean (standard deviation [SD]) age of 36.8 (6.5) years (range: 26–47 years), mean (SD) body weight of 87.8 (18.9) kg, and body mass index that ranged from 19.0 to 37.6 kg/m ^2^ . The median (range) total number of bleeding episodes in the 12 months before screening was 36 (2–80), and most participants ( *n* = 10; 76.9%) were receiving on-demand treatment. All participants completed the study treatment period and were included in the pharmacokinetic analysis.

**Fig. 1:**
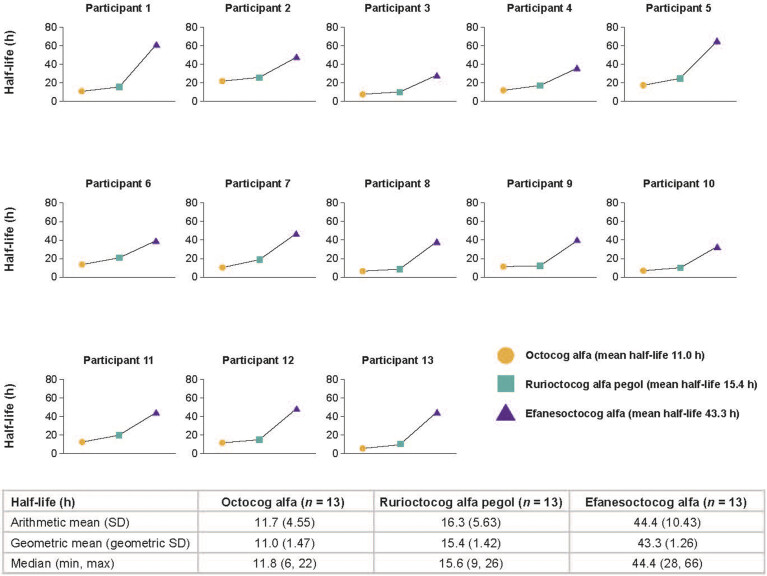
Individual participant half-life profiles for octocog alfa, rurioctocog alfa pegol, and efanesoctocog alfa treatments. Pharmacokinetic parameters were derived using baseline-corrected FVIII activity that was estimated by the one-stage activated partial thromboplastin time–based clotting assay. Pharmacokinetic sampling was performed over a period of 3, 5, and 14 days after the administration of octocog alfa, rurioctocog alfa pegol, and efanesoctocog alfa, respectively. FVIII, factor VIII; h, hour; SD, standard deviation.

**Fig. 2:**
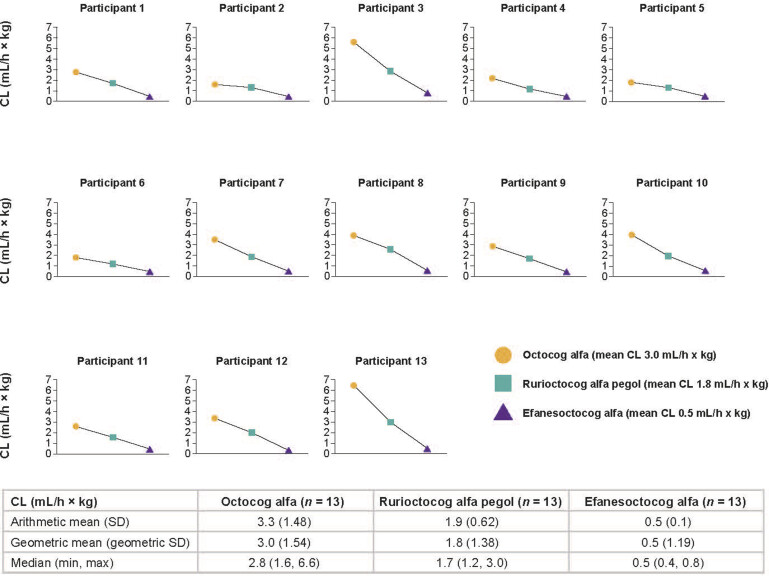
Individual participant clearance profiles for octocog alfa, rurioctocog alfa pegol, and efanesoctocog alfa treatments. Pharmacokinetic parameters were derived using baseline-corrected FVIII activity that was estimated by the one-stage activated partial thromboplastin time–based clotting assay. Pharmacokinetic sampling was performed over a period of 3, 5, and 14 days after the administration of octocog alfa, rurioctocog alfa pegol, and efanesoctocog alfa, respectively. CL, clearance; FVIII, factor VIII; h, hour; SD, standard deviation.

**Fig. 3:**
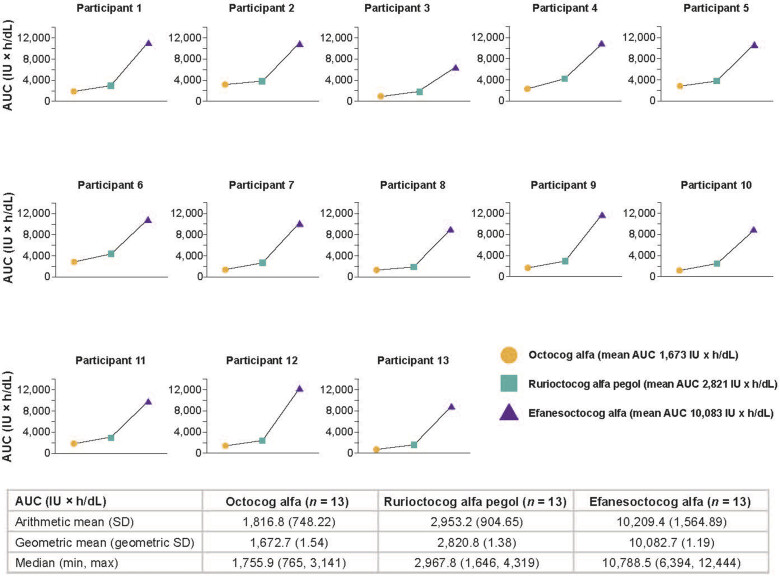
Individual participant AUC profiles for octocog alfa, rurioctocog alfa pegol, and efanesoctocog alfa treatments. Pharmacokinetic parameters were derived using baseline-corrected FVIII activity that was estimated by the one-stage activated partial thromboplastin time–based clotting assay. Pharmacokinetic sampling was performed over a period of 3, 5, and 14 days after the administration of octocog alfa, rurioctocog alfa pegol, and efanesoctocog alfa, respectively. AUC, area under the curve; FVIII, factor VIII; IU, international unit; h, hour; SD, standard deviation.

**Fig. 4:**
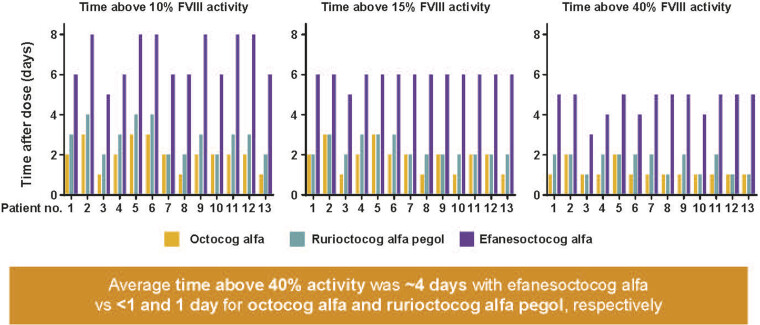
Time above 10%, 15%, and 40% FVIII activity profiles for all participants receiving octocog alfa, rurioctocog alfa pegol, and efanesoctocog alfa. Baseline-corrected FVIII activity was estimated by the one-stage activated partial thromboplastin time–based clotting assay. Pharmacokinetic sampling was performed over a period of 3, 5, and 14 days after the administration of 50 IU/kg doses of octocog alfa, rurioctocog alfa pegol, and efanesoctocog alfa, respectively. The FVIII activity for all three treatments was assessed at 10 min (± 2 min), 30 min (± 5 min), 1 h (± 10 min), 6 h (± 10 min), 24 h (± 1 h), 48 h (± 2 h), and 72 h (± 2 h) following dosing. Rurioctocog alfa pegol FVIII activity was additionally assessed at 96 h (± 2 h) and 120 h (± 2 h) following dosing and efanesoctocog alfa FVIII activity was additionally assessed at 96 h (± 2 h), 120 h (± 2 h), 168 h (± 2 h), 240 h (± 2 h), 288 h (± 2 h), 336 h (± 24 h), and 28 days (± 5 days) after dosing. FVIII, factor VIII; h, hour; min, minute; IU, international unit.

In this post hoc study, the pharmacokinetic profiles in participants who received a single 50 IU/kg dose of efanesoctocog alfa demonstrated a clear and consistent increase in half-life that was evident in all individuals ( Fig. 1 ). Geometric mean (geometric SD) values, as reported previously, 9 were 43.3 (1.26) hours for efanesoctocog alfa, 11.0 (1.47) hours for octocog alfa, and 15.4 (1.42) hours for rurioctocog alfa pegol. Similarly, clearance of efanesoctocog alfa was markedly and consistently lower than that of octocog alfa and rurioctocog alfa pegol for all individuals ( Fig. 2 ). Geometric mean (geometric SD) values for the whole group were 0.5 (1.19) mL/h × kg for efanesoctocog alfa, 3.0 (1.54) mL/h × kg for octocog alfa, and 1.8 (1.38) mL/h × kg for rurioctocog alfa pegol. Exposure (as measured by AUC _inf_ ) was also notably higher for efanesoctocog alfa versus octocog alfa or rurioctocog alfa pegol in all individual participants, and when groups were assessed as a whole, as reflected by geometric mean (geometric SD) values of 10,082.7 (1.19), 1,672.7 (1.54), and 2,820.8 (1.38) IU × h/dL, respectively ( Fig. 3 ). In addition, all participants experienced more days above 10, 15, and 40% FVIII activity after treatment with efanesoctocog alfa compared with octocog alfa or rurioctocog alfa pegol ( Fig. 4 ). Results corroborate pharmacokinetic data from individuals ( *n* = 17) receiving once-weekly prophylaxis with efanesoctocog alfa (50 IU/kg) in the phase 3 registration study, which reported a cohort-level geometric mean half-life of 47 hours and steady-state clearance of 0.439 mL/h/kg. 8

Endogenous VWF acts as a chaperone for FVIII, protecting it from degradation and clearance, 15 but in doing so imposes a VWF half-life ceiling on FVIII activity. 16 Indeed, because the clearance of PEGylated, Fc fusion, and single-chain FVIII replacement therapies remains largely dependent on VWF, the half-lives of these products are only moderately increased compared with SHL products. 3 Because of the effects of many physiologic contributory factors (including body weight, age, diet, ABO antigen status, vascular integrity, and inflammatory state) in addition to differences in receptor expression, sequence variations, polymorphisms, mutations, and glycosylation variations for VWF and other associated proteins, 3 interindividual VWF clearance is known to vary significantly. 14 Results from a previous post hoc analysis 17 of data from 38 adult males enrolled in either the phase 1/2a EXTEN-A study 6 or a phase 1 repeat-dose study 18 demonstrated that after both single and repeat dosing, the half-life and clearance of efanesoctocog alfa were independent of pre-dose VWF levels. 17 Further, a post hoc analysis of data from the XTEND-Kids study revealed that efanesoctocog alfa pharmacokinetic parameters, including half-life and clearance, were unaffected by blood group. 19 These observations are supported by results of a population pharmacokinetic study of efanesoctocog alfa in patients with severe hemophilia A 20 which, using a linear one-compartment model, revealed that baseline VWF level was not a statistically significant covariate.

In conclusion, the results of this post hoc analysis of data from a comparative phase 1 sequential pharmacokinetic study confirm that the longer half-life, lower clearance, and greater exposure (AUC _0-inf_ ) achieved after treatment with efanesoctocog alfa versus octocog alfa or rurioctocog alfa pegol are consistently observed at the individual participant level. In addition, all enrolled participants had more days above 10, 15, and 40% FVIII activity level after treatment with efanesoctocog alfa compared with octocog alfa or rurioctocog alfa pegol. These results add to the existing literature for efanesoctocog alfa as a treatment that can reliably provide protective FVIII levels with low interindividual variability.
